# Investigating the Effect of High-Voltage Electrostatic Field (HVEF) Treatment on the Physicochemical Characteristics, Bioactive Substances Content, and Shelf Life of Tomatoes

**DOI:** 10.3390/foods13172823

**Published:** 2024-09-05

**Authors:** Xiaobao Nie, Zhijie Zuo, Li Zhou, Zhe Gao, Lilin Cheng, Xiaoli Wang, Linghong Nie, Ping-Hsiu Huang

**Affiliations:** 1 School of Life Science and Food Engineering, Huaiyin Institute of Technology, Huai’an 223003, China; zzj123452022@163.com (Z.Z.); m19825570635@163.com (L.Z.); chengll0816@163.com (L.C.); xlwang@hyit.edu.cn (X.W.); haitaonie@163.com (L.N.); 2 College of Food Science and Technology, Hebei Agricultural University, Baoding 071000, China; gaozhe1979@sina.com; 3 School of Food, Jiangsu Food and Pharmaceutical Science College, Huai’an 223003, China; hugh0530@gmail.com

**Keywords:** high-voltage electrostatic field (HVEF), shelf life, tomato, postharvest quality, bioactive compounds

## Abstract

This study evaluated the ability of a high-voltage electrostatic field (HVEF) treatment to extend the shelf life of tomatoes. Tomatoes were exposed to HVEF treatment for different lengths of time, and the physicochemical properties of tomatoes and bioactive compounds were monitored during 28 days of storage at 4 °C. The results indicated that the quality parameters of tomatoes were better maintained during storage by the HVEF treatment relative to the control treatment, extending their shelf life by 14–28 days. The HVEF treatment mitigated losses in firmness, weight, color changes, and bioactive substances, such as total phenolic content, total flavonoid content, ascorbic acid, and lycopene. The activity of pectin-degrading enzymes was also inhibited. The best exposure times for the HVEF treatment were 90 and 120 min. While the measured parameters decreased in both the control and HVEF treatment groups, the decrease in all of these measured parameters was significantly less (*p* < 0.05) in the optimum HVEF treatment groups than in the control. While the physicochemical properties may vary between different tomato varieties, the HVEF treatment of harvested tomatoes for 90 or 120 min can mitigate the degradation of quality parameters and loss of bioactive compounds incurred during the postharvest storage of tomatoes, thus maintaining their commercial value.

## 1. Introduction

Recent studies have reported that magnetic and electrical fields, particularly HVEFs, can effectively maintain the shelf life of produce by inhibiting oxidative browning and tissue softening, maintaining product quality parameters, and inhibiting microbial growth [1,2,3]. In addition, HVEFs have been reported to induce the perforation of plant cell membranes, which stimulates the synthesis of secondary metabolites, thus enhancing tolerance to abiotic and biotic stresses and increasing the content of bioactive substances such as vitamin C, carotenoids, and phenolics, which increases antioxidant capacity [4,5]. The HVEF is classified as a nonthermal, low-energy treatment that produces electrical wind through a corona discharge between at least two electrodes [3,6,7]. HVEF treatment is particularly applicable as a postharvest treatment to fruits and vegetables to preserve firmness, sensory quality parameters, and nutritional composition [5,8,9]. However, the limitations and challenges of high investment costs, adjustment of the process parameters, and the immaturity of HVEF technology have not been adequately addressed [5].

Most fruits and vegetables are marketed globally so that they will be available to consumers year-round. Therefore, the ability to extend their shelf life and maintain their quality as well as their nutraceutical content is essential [10]. Various parameters, including transpiration, ripening, and spoilage, affect the postharvest quality of fruits and vegetables. Notably, the rapid increase in the rate of respiration and ethylene generation that occurs in climacteric fruits results in faster ripening, senescence, and product deterioration [5,11,12,13].

The beneficial effects of HVEF corona discharge are the result of the ionization of air within the environment, which produces ozone, negative air ions, and other molecules (e.g., free radicals). These molecules inhibit or kill pathogenic microorganisms present on harvested fruits and vegetables, reduce the stomatal opening of epidermal cells, and alter sugar metabolism [14,15].

Tomatoes are a low-calorie vegetable that is rich in nutrients (lycopene; vitamins A, C, and E; and minerals) that has significant commercial value, and it has been extensively studied with respect to all of its aspects [16,17]. Unfortunately, tomatoes rapidly experience senescence during their processing, distribution, and marketing and lose their quality and commercial value. Tomatoes are also susceptible to chilling injury when exposed to temperatures below 10–13 °C and cannot be stored for more than 7 days under high temperatures and environmental conditions present in summer months [18,19,20]. Regrettably, chemical preservatives such as chlorinated compounds used to extend the shelf life of fruits for prolonged periods can readily react with other compounds to produce toxic and harmful substances that are hazardous to human health. Therefore, the development of safe, minimal, eco-friendly processing methods is urgently needed [21,22,23]. Technologies used during the postharvest processing of fruits and vegetables inhibit and/or prevent the establishment of pathogenic and foodborne microorganisms, assuring food safety and also help to maintain functional attributes, such as nutrient and nutraceutical content, as well as quality parameters, including taste, color, texture, and appearance. Current methods of postharvest processing of produce, however, are often criticized due to concerns about their potential negative impact on human health and the environment [10,24]. Therefore, there is a need to develop new approaches to protect and maintain the quality of cold-sensitive commodities, such as tomatoes, during storage and transportation [12,19]. To address this need, the present study characterized changes in the physicochemical properties of tomatoes during storage for 28 days at 4 °C following exposure to an HVEF treatment for varying lengths of time (30, 60, 90, and 120 min). The overall objective of the study was to determine if HVEF treatment could be used to delay losses in quality indicators and extend the shelf life of tomatoes.

## 2. Materials and Methods

### 2.1. Materials

Beefsteak tomatoes (*Solanum lycopersicum*) were harvested at commercial maturity in August 2023 from a farm located in the Huaiyin District, Huai’an, China. The tomatoes were selected based on size, color, and ripeness. All chemicals were purchased from Sigma-Aldrich (Merck KGaA, Darmstadt, Germany).

### 2.2. HVEF Treatment

The HVEF protocol utilized in the present study was as described by Liu et al. [25]. The AP-HVEF equipment consisted of a DC electrostatic generator (SC-PME 50, COSMI, Co., Denba Co., Ltd., Jiaxing, China) with an output voltage of 50 kV, while the two-layer plate device used to generate a current for the HVEF had a shelf spacing of 8 cm, output voltage of 48 kV, and generated an electric field strength of 600 kV/m. Groups of thirty tomatoes were placed between the two flat plates and exposed to an electric current. Four treatment times were utilized (30, 60, 90, and 120 min). After HVEF treatment, the tomatoes were placed in storage at 4 °C for 28 days, and physicochemical properties and quality indicators were assessed every 7 days. The control group comprised tomatoes not treated with HVEF. Photos were taken regularly to document changes in the appearance of tomatoes in the control and HVEF treatment groups.

### 2.3. Firmness 

Tomato firmness was assessed in Newtons (N) as described in [25,26]. Firmness was measured using a texture analyzer (TA-XT2, Stable Micro Systems Ltd., Godalming, UK). The conditions were as follows: A TA39 probe was used, and samples were compressed at 5 mm/s to 25% of the original height on the side of each tomato fruit. Firmness was measured at three random locations along the equator of each fruit. Firmness values are expressed in Newtons (N).

### 2.4. Weight Loss

Fresh weight loss in tomatoes was measured as described by Zhao et al. [5]. The cumulative loss in fresh weight was measured in the HVEF and control treatment groups of tomatoes during the storage. Weight loss was calculated using the following formula:

Weight loss (%) = [(Initial weight of tomato − Weight of tomato by a specific storage time)/Initial weight of tomato] × 100

### 2.5. Color Analysis and Appearance 

Color changes in tomatoes during storage were determined as described in Hou et al. [27]. *L* (brightness), *a* (redness–greenness), and *b* (yellowness–blueness) values were determined using a color meter (ZE 2000, Nippon Denshoku Industries Co., Ltd., Tokyo, Japan). A standard white calibration plate was used to calibrate the colorimeter. The appearance of tomatoes in the control and HVEF 120 min treatment groups was recorded using a digital camera (EX-FH20, 20 megapixels, Casio Computer Co., Ltd., Tokyo, Japan) at 7, 14, 21, and 28 days of storage.

### 2.6. Pectin Content 

Pectin content was determined using the protocol reported by Wu et al. [28]. Ten grams of tomato tissue were homogenized in 100 mL of deionized water for 2 min using a sterile homogenizer. The pH of the homogenate was then adjusted to pH 2.0 with 1N HCl, followed by heating at 80 °C for 1 h while stirring to extract the pectin. After cooling to 25 °C, the pulp residue was filtered, and the resulting filtrate was added to an equal volume of 95% ethanol (*v*/*v*) and centrifuged at 3000× *g* for 10 min at 4 °C. Next, the resulting pellet was then added to the same volume of 75% ethanol (*v*/*v*), and the above extraction procedure was repeated 2 times to obtain alcohol insoluble solids (AISs) from each tomato sample. Pre-cooled H_2_SO_4_ (2 mL) in 15 mL of distilled water was slowly added to 5 mg of tomato AIS, followed by stirring in an ice bath for 1 h to obtain a pectin solution. Subsequently, 0.5 mL of pectin solution was mixed with 3 mL of 12.5 mM sodium tetraborate in sulfuric acid in an ice bath. The sample was then heated in a boiling water bath for 5 min, cooled, and then mixed with 0.05 mL of 0.15% m-hydroxy biphenyl solution (0.5% NaOH) and left for 5 min. Absorbance of the test solution was measured at 520 nm. A standard curve was constructed using d-galacturonic acid to calculate pectin content in each of the samples.

### 2.7. Enzyme Activity of Pectin Methylesterase (PME) and Polygalacturonase (PG)

PME [29] and PG activities [28] were determined. Ten grams of tomato tissue were homogenized in 15 mL of 100 mM phosphate buffer (pH 6.2) containing 1 mM EDTA and 1% (*w*/*v*) polyvinyl pyrrolidine (PVP). Each sample was homogenized for 2 min and then centrifuged at 12,000× *g* for 20 min at 4 °C. Next, 0.2 mL of supernatant was added to 0.8 mL of 0.3% citrus pectin (degree of methyl esterification: DE 60–66%) in 0.2 M acetate buffer (pH 4.0). The reaction mixture was then incubated in a water bath at 37 °C for 30 min, after which the reaction was terminated by placing the sample in a boiling water bath for 5 min. For PG activity determination, 1 mL of 3,5-dinitrosalicyclic acid (DNS) solution was added and boiled for 10 min to determine the production of the reducing group, whereas for PME activity determination, this mixture was then titrated with sodium hydroxide. The absorbance of the samples was measured at 510 nm for PG activity. PG activity units were defined as the amount of enzyme catalyzing the release of 1 μmol galacturonic acid per min at 37 °C, whereas PME activity units were defined as the amount of PME consuming 1 μmol sodium hydroxide per min.

PE activity was measured by adding 100 μL of the same supernatant obtained from the phosphate buffer extraction previously indicated to 2 mL of 0.5% pectin, 0.2 mL of 0.01% bromothymol blue, and 0.7 mL of dd water. The obtained solution was then placed in a water bath at 40 °C for 20 min. After cooling to room temperature, the absorbance value of the solution was measured at 620 nm and then measured again after 20 min to calculate PE activity. One unit of enzyme activity (U/mg protein) was designated as the amount of enzyme required to produce 1 μg of free carboxyl per min by PE hydrolysis at 25 °C.

### 2.8. Total Phenolic Content (TPC) and Total Flavonoid Content (TFC)

TPC was determined as described by Li et al. [30] and Nkolisa et al. [31]. One gram of tomato tissue was homogenized in 1 mL of Folin–Ciocalteu reagent. Subsequently, 2 mL of 20% Na_2_CO_3_ was added, the solution was thoroughly mixed, and the absorbance of the solution was measured at 750 nm. A standard curve was prepared using gallic acid (GA) and used to calculate total phenolic content in the sample solution, which was expressed as GA equivalents (mg GAE/g).

TFC was determined as described by Li et al. [32] and Nkolisa et al. [31]. One gram of tomato tissue was homogenized in 0.3 mL of 5% NaNO_2_ and 0.3 mL of 10% AlCl_3_ and allowed incubate for 5 min. Then, 2 mL of 1M NaOH was added, and the volume of the solution was adjusted to 10 mL with distilled water, mixed uniformly, and incubated at room temperature for 15 min. Subsequently, the absorbance of the test solution was measured at 510 nm. A standard curve was constructed using quercetin and used to calculate total flavonoid content in the sample solution. TFC is expressed as quercetin equivalents (mg QE/g).

### 2.9. Vitamin C and Lycopene Content

Vitamin C content was determined using the indophenol method as described in [33]. Ascorbic acid (50 mg) was mixed with 50 mL of metaphosphoric acid-acetic acid to obtain a standard solution. Then, 2.0 mL of the ascorbic acid standard solution using the indophenol solution was prepared by dissolving 42 mg of sodium bicarbonate and 50 mg of 2,6-dichloroindophenol sodium salt in 50 mL of distilled water and adjusting the final volume to 200 mL with distilled water. This solution was refrigerated and kept in the dark until use. Titration of the standard solution and the test solution was conducted, and the volume needed to reach an endpoint of the reaction was recorded. The endpoint of the reaction was defined as when the solution took on a distinct rose pink color for 5 s. Ascorbic acid content in the samples (mg/100 g) was calculated using the following formula:

Ascorbic acid (Vc, mg/100 g) = (Sample titration − blank titration) × (titer of indophenol solution/2 mL) × volume consumed by titration of the sample

Lycopene content was determined using the protocol described in [31,34]. A total of 1.2 mL of tomato homogenate was mixed with 10 mL each of 0.05% BHT, 95% ethanol, and hexane, stirred, and then incubated in an ice bath for 15 min. Then, 6 mL of distilled water was added to the sample solution, stirred, incubated for another 5 min over ice, and then allowed to stand for 15 min. Absorbance of the supernatant was measured at 503 nm. All operations were performed under dark conditions. Lycopene content was calculated using the following equation:

Lycopene content (mg/100 g) = Abs (503 nm) × 137.4 (lycopene constant coefficient)

### 2.10. Statistical Analysis

The values presented in the tables represent the mean ± standard deviation (SD). Every assay was conducted in triplicate (*n* = 3). All data were subjected to a two-way analysis of variance (ANOVA) followed by a Duncan’s multiple range test to determine significant differences (*p* < 0.05) within treatment groups at the different sampled timepoints and between all treatment groups and time points using IBM SPSS statistical software (version 26.0) (IBM Co., Armonk, NY, USA).

## 3. Results and Discussion

### 3.1. Changes in Firmness

Firmness is an important quality indicator of fresh produce [35]. The effect of the HVEF treatments on tomato firmness are reported in Table 1. The firmness of tomatoes in three of the HVEF treatment groups (60, 90, and 120 min) exhibited a slight but significant decrease (*p* < 0.05) at 0 d of storage, relative to the control and 30 min HVEF treatment groups. The 30 min HVEF treatment group exhibited the highest significant decrease (*p* < 0.05) in firmness relative to the 60, 90, and 120 min HVEF treatment groups on days 14–28. Notably, tomatoes exposed to the HVEF for 90 and 120 min maintained the highest level of firmness during the period of 21–28 days of storage, with values that were significantly higher (*p* < 0.05) than in the other HVEF and control treatment groups. These results are consistent with previous studies [5,36] on the effect of HVEF treatments on produce firmness. In this regard, HVEF treatment has been reported to inhibit the activity of cell wall-degrading enzymes, such as pectinase and cellulase [35,37]. Our results indicate that the 90 and 120 min HVEF treatment effectively mitigated the reduction in firmness that occurs in tomatoes during postharvest storage. 

### 3.2. Changes in Fresh Weight Loss 

Weight loss in fruits during storage is the result of respiration, nutrient depletion, and the water vapor pressure gradient that exists between the tomato tissue and the surrounding air [1,5]. The degree of fresh weight loss is also affected by the temperature and relative humidity of the storage conditions [38]. Data on the effect of HVEF treatment on fresh weight loss in tomatoes during storage is presented in Table 1. Our results indicated that tomatoes exposed to the HVEF treatments exhibited a slight reduction on day 0 relative to the control. A positive association of percentage weight loss with storage time was observed in all treatment groups. The smallest reduction in fresh weight with storage time was observed in the 90 and 120 min HVEF treatment groups, especially during the latter period of storage (days 14–28); however, the percentage fresh weight loss in all HVEF treatment groups was significantly lower (*p* < 0.05) than the percentage weight loss in the control. These results are again consistent with previous results reported by Zhao et al. [5]. Significant increases (*p* < 0.05) in percentage weight loss were observed between each of the sampled timepoints in all treatment groups. Significant differences (*p* < 0.05) in the percentage weight loss in the 90 and 120 min HVEF treatment groups and all other treatment groups were observed beginning at 7 d of storage. One plausible explanation that has been proposed for the lower rates of weight loss in the HVEF treatment groups is that the HVEF treatment may modify the transmembrane potential of plant cells, resulting in the directional movement of charged ions on both sides of the membrane, thus generating bioelectric currents that promote biochemical reactions and alter physiological processes that affect cellular metabolism [5,7,39]. In this regard, membrane potential differences have been reported to increase in response to HVEF exposure [5]. While the exact mechanism will require further investigation, our results indicate that the HVEF treatments clearly reduced the percentage weight loss in tomatoes during storage, thus extending their potential shelf life. 

### 3.3. Color Analysis and Physical Appearance

Color and appearance are key factors in a consumer’s evaluation of produce quality and when making purchase decisions [13,40]. Data on the color evaluation obtained in our study are presented in Table 1. The results indicated that no differences in the *L* value were observed between any of the treatment groups, including the control, on day 0 of storage. However, significant differences (*p* < 0.05) appeared between the HVEF treatment groups (except the 120 min HVEF treatment group) and the control as storage time increased. More specifically, *L* values in the other HVEF treatment groups (30, 60, and 90 points) did not differ significantly from their initial values as storage time progressed, but they were significantly different (*p* < 0.05) from the control and 120 min HVEF treatment group. These results are consistent with previous published reports on the effect of HVEF treatment on tomato [5,41]. 

All treatment groups exhibited an increasing trend in *a* value with storage time, while *b* values exhibited a decreasing trend. No significant differences (*p* < 0.05) were observed between any of the treatment groups at any specific timepoint of storage. Variations in color parameters during storage can be attributed to differences in lycopene content. In this regard, Jia er al. [41] attributed differences in color in stored tomatoes to the effect of HVEF treatment on inhibiting the rate of respiration. 

**Figure 1:**
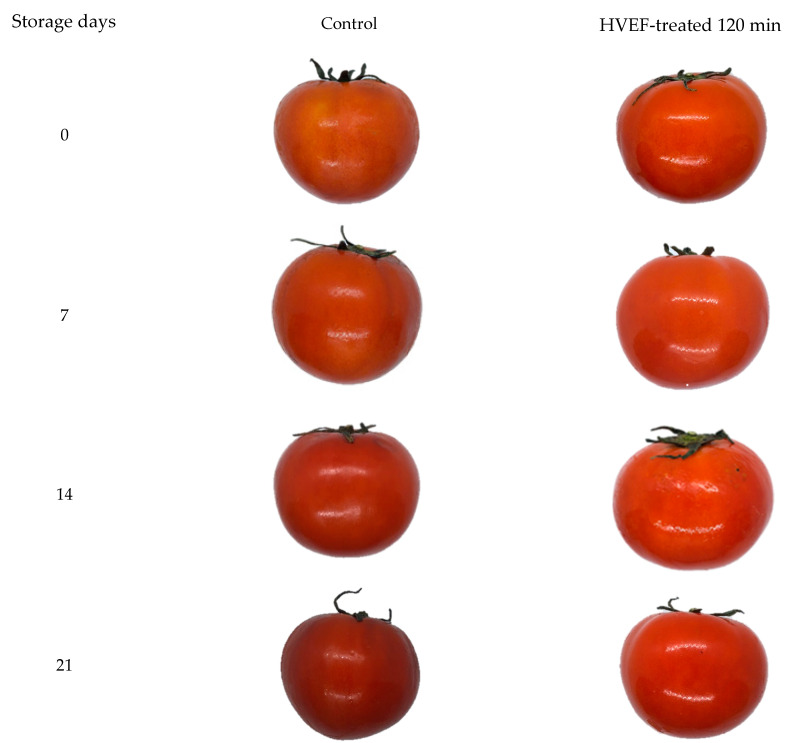

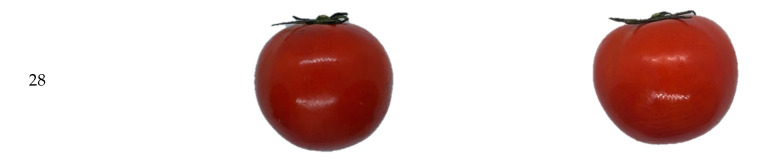
Representative photographs of changes in the appearance of tomatoes from the control and 120 min HVEF treatment groups during 28 days of storage at 4 °C.

Regarding their visual appearance, tomatoes in the control and 120 min HVEF treatment groups exhibited a significantly darker appearance, relative to the other HVEF treatment groups, with the control group exhibiting the darkest color and a slightly wrinkled skin (Figure 1). The collective data in our study provide evidence that the HVEF treatments (except the 120 min HVEF treatment) maintained the initial color of tomato color during subsequent storage at 4 °C. In this regard, HVEF treatments of tomato have been previously reported to affect both the respiration rate and pigment content (chlorophyll, carotene, hydroxylated carotenoids, lycopene, and other pigments) during the postharvest ripening process [5,41,42].

### 3.4. Pectin Content 

Pectin is a structural polysaccharide in the cell wall that polymerizes and limits the access of cell wall-degrading enzymes, thus increasing the resistance of cell walls to biodegradation. During the ripening process, cell wall-degrading enzymes (pectinases, cellulases, and hemicellulases) begin to breakdown the cross-linking that exists between different cell wall components and degrade the actual structural carbohydrates, resulting in softening of the fruit and increasing its susceptibility to decay [43]. In the present study, no differences in pectin content were observed between any of the HVEF treatment groups and the control on day 0 of storage (Table 2). Subsequently, a decreasing pattern in pectin content was observed beginning on day 7 of storage with the control treatment group, exhibiting the greatest decrease (*p* < 0.05) in pectin content. While pectin content did decrease with storage time in all HVEF treatment groups, no significant differences (*p* < 0.05) were observed between the different HVEF treatment groups until 28 d of storage. Comparatively, pectin content in the control on day 14 was similar or less than the pectin content in all HVEF treatment groups on day 28 of storage. A correlation between decreases in pectin content, firmness, and weight loss has been previously reported [11,20].

### 3.5. Pectic Enzymes Activity (PME and PG) 

Pectin degradation during the softening process in fruits is typically catalyzed by the synergistic action of polygalacturonase (PG), pectin methylesterase (PME), pectin lyase (PL), and β-galactosidase (β-gal) [11,28,29,43]. The results of our study revealed no significant differences (*p* < 0.05) in PG and PME activity between any of the treatment groups on day 0 (Table 2). However, the activity of both enzymes exhibited an increasing trend with storage time. The control treatment group exhibited the highest increase in PG activity at 14 d of storage followed by the 30 min HVEF treatment group. PG activity in both these groups was significantly higher (*p* < 0.05) than PG activity in the other HVEF treatment groups. The greatest inhibition of PG activity was observed in the 90 and 120 min HVEF treatment groups. 

A slight increase in PME activity was observed from 0 to 14 d of storage with small but significant differences (*p* < 0.05) being observed between the control and HVEF treatment groups (Table 2). Notably, the control group experienced the highest increase in PME activity among the treatment groups from days 21 to 28 of storage, with PME activity being significantly higher (*p* < 0.05) than in all of the HVEF treatment groups. These results indicate that the HVEF treatments inhibit both PG and PME activity in tomatoes during storage. This fact was evidenced by the higher firmness and lower weight loss observed in the HVEF treatment group during storage relative to the control group of tomatoes. The mechanism by which HVEF treatment affected enzyme activity may have been due to its impact on the expression level of genes encoding PG or PME, it increasing the degradation of the enzymes, or it simply reducing enzyme activity by decreasing the rate of metabolism. We speculate that the increase in PG activity at the lower doses of HVEF treatment may have been due to the activation of a stress-related response, while the decreased levels of PG activity at the higher doses was due to the overall impact of HVEF treatment on metabolic rates. 

### 3.6. Total Phenolic Content (TPC) and Total Flavonoid Content (TFC)

Various technologies commonly used in the fruit industry affect the phenolic content of the product [10]. The measurement of secondary metabolites, including TPC, TFC, ascorbic acid, and lycopene, have been recognized as indicators of nutritional quality in different horticultural products [31,44]. No significant differences were observed in TPC between the control and HVEF treatment groups on day 0 of storage (Table 3). The control group, however, exhibited a slight increase in TPC on day 7 of storage, followed by a significant decreasing trend (*p* < 0.05) from days 14 to 28 of storage. In contrast, tomatoes in the HVEF treatment groups exhibited a slight increase in TPC after 14 d of storage, followed by a decreasing trend from days 21–28 of storage. Decreases in TPC in tomatoes have been suggested to be due to ripening, phenolic-protein binding, or other chemical structural changes [31,45,46]. In general, however, the decline in TPC in the HVEF treatment groups was delayed by approximately seven days relative to the control treatment group. This delay represents an extension in quality that would benefit retailers and enterprises that bulk process fresh tomatoes. In addition to the HVEF treatment, storage temperature and humidity should be investigated as factors that impact TPC. In this regard, the TPC of fruits stored at 10 °C has been reported to be significantly higher than it is in fruit stored at 5 °C, while an adequate TPC level was observed during the entire storage period in fruit stored at 0 °C [47].

No differences in TFC were observed between the control and HVEF treatment groups on day 0 of storage (Table 3). TFC exhibited a decreasing trend during storage in all treatment groups; however, the greatest decrease in TFC during storage was observed in the control group of tomatoes. TFC in all of the HVEF treatment groups also decreased with storage time, but the decrease was moderate compared with the control group of tomatoes. The highest TFC after 28 d of storage was observed in the 90 and 120 min HVEF treatment groups, with TFC levels of 5.07 ± 0.05 and 5.18 ± 0.04 (mg QE/g), respectively. Several previous studies have reported that a variety of abiotic stresses, including high-intensity ultrasound, pulsed electric fields, and high-intensity UV, can induce the synthesis of phenylalanine amylolytic enzymes, resulting in an increase in TPC and TFC [35,48,49].

### 3.7. Ascorbic Acid and Lycopene Content 

Ascorbic acid content exhibited a negative relationship (*p* < 0.05) with storage time in all treatment groups in our study (Table 3). Previous studies have reported that vitamin C content can vary, wherein both increases due to maturation and decreases due to high respiration rates, oxidative degradation, and carbon dioxide accumulation can occur [5,42,50]. Fruits and vegetables exposed to a constant low temperature (12 °C) have been reported to have reduced rates of respiration, metabolism, senescence, and decay development [11,31,38,51], while the opposite occurs in produce stored at 25 °C or higher [47]. In contrast to these reported results, a moderate decrease in vitamin C content was observed in all HEVF treatment groups, relative to the control group. Notably, ascorbic acid content after 7 days of storage in the 90 and 120 HVEF treatment groups was similar to the initial content. While ascorbic acid content decreased in all of the treatment groups during storage, tomatoes in all HVEF treatment groups had a significantly higher (*p* < 0.05) level of ascorbic acid than the control treatment group after 28 days of storage. These data indicate that HVEF treatment mitigated the impact of the maturation process on vitamin C content. Similar results in tomatoes have been reported by Zhao et al. [5].

The pattern of lycopene content in tomatoes during storage was similar to the pattern described for ascorbic acid. The highest lycopene content (14.20 mg/100 g) after 28 days of storage was observed in the 120 min HVEF treatment group and was significantly higher (*p* < 0.05) than all of the other treatment groups (Table 3). Our data indicate that the HVEF treatment mitigated the decline in lycopene content during storage, especially during the period of 7–14 days of storage. It should be noted, however, that patterns of increasing or decreasing lycopene content over time may be due to the characteristics of different varieties [52].

González-Casado et al. [4] reported that tomato fruit treated with a pulsed electric field (PEF) exhibited an increase in lycopene content and that the increase was correlated with electric field intensity (0.4 to 2 kV cm^−1^). This response was attributed to the enhancement of secondary metabolite metabolism induced by the PEF treatment. Electric field treatments have also been reported to induce both reversible and irreversible pore formation in cell membranes, a process referred to as electro-osmosis [4,53]. This would lead to the leakage of bioactive components, such as carotenoids and lycopene, from tomato cells into the extracellular environment. Electro-osmosis also has a substantial impact on the texture and color of tomatoes [4].

## 4. Conclusions

In our study, tomatoes were exposed to HVEF (600 kV/m) treatment for 30, 60, 90, or 120 min prior to being placed in cold storage at 4 °C for 28 days. The study was conducted to determine if an HVEF treatment could be applied prior to distribution to obtain retail products with extended shelf life and enhanced nutritional-related properties. Several physicochemical quality indicators were assessed on a weekly basis, including firmness, weight loss, color and appearance, pectin-degrading enzyme activity (PG and PME), TPC, TFC, ascorbic acid, and lycopene. All of these parameters were significantly impacted (*p* < 0.05) in a positive manner by the HVEF treatments during the 28 d of storage relative to untreated, control tomatoes. Despite some minor negative impacts in the color and appearance and bioactive substance content in some of the HVEF treatment groups (primarily the 120 min HVEF treatment group) after 28 days of storage, we suggest that HVEF treatments can be used to extend the shelf life of tomatoes by as much as 21 days of storage. This would greatly benefit retailers by increasing the time of marketability, thus reducing economic losses. Our study provides vital information for the use of HVEF treatment to extend the shelf life of tomatoes. Importantly, HVEF studies need to be conducted on a variety of other tomato varieties to determine appropriate HVEF intensity levels and durations. Furthermore, it may be possible to combine HVEFs with other processing strategies such as controlled atmosphere packaging and an appropriate temperature control to further extend the shelf life of tomatoes. Additionally, further studies should be conducted to develop a detailed understanding of the impact of HVEF treatment on fruit metabolism and structure. Moreover, another challenge that needs to be addressed is determining how to apply HVEF technology on fruits that are highly conductive to ensure that HVEF treatments are beneficial and achieve the objectives of extending the shelf life of produce while preserving their quality and nutritional value.

## Figures and Tables

**Table 1:** Effect of different HVEF (600 kV/m) treatment times (30, 60, 90, and 120 min) on firmness, weight loss, and color (*L*, *a*, and *b* values) in tomatoes stored for 28 days at 4 °C.

	Storage Time (Days)	Control Group	High-Voltage Electrostatic Field (HVEF) Treatment with 600 kV/m			
			30 min	60 min	90 min	120 min
Firmness (N)	0	8.43 ± 0.07 ^aA^	8.40 ± 0.07 ^aA^	8.21 ± 0.06 ^aB^	8.17 ± 0.04 ^aB^	8.12 ± 0.03 ^aC^
	7	7.74 ± 0.09 ^bA^	8.13 ± 0.04 ^bB^	8.01 ± 0.08 ^bB^	8.08 ± 0.03 ^bB^	8.06 ± 0.03 ^aB^
	14	7.50 ± 0.11 ^cA^	7.76 ± 0.11 ^cB^	7.86 ± 0.04 ^cB^	7.95 ± 0.03 ^bB^	7.95 ± 0.02 ^bB^
	21	6.83 ± 0.11 ^dA^	7.35 ± 0.10 ^dB^	7.58 ± 0.04 ^dC^	7.74 ± 0.06 ^cD^	7.81 ± 0.03 ^cD^
	28	6.17 ± 0.05 ^eA^	7.17 ± 0.07 ^eB^	7.35 ± 0.06 ^eC^	7.55 ± 0.06 ^dD^	7.54 ± 0.11 ^dD^
Weight loss (%)	0	0.00 ± 0.00 ^aA^	0.07 ± 0.02 ^aB^	0.13 ± 0.01 ^aC^	0.16 ± 0.01 ^aD^	0.18 ± 0.02 ^aD^
	7	3.23 ± 0.03 ^bA^	3.18 ± 0.05 ^bB^	3.08 ± 0.06 ^bC^	2.83 ± 0.06 ^bD^	2.71 ± 0.02 ^bD^
	14	4.29 ± 0.08 ^cA^	4.13 ± 0.04 ^cB^	4.11 ± 0.05 ^cB^	3.89 ± 0.07 ^cC^	3.41 ± 0.04 ^cD^
	21	6.51 ± 0.04 ^dA^	6.24 ± 0.08 ^dB^	6.15 ± 0.06 ^dB^	6.05 ± 0.05 ^dC^	5.67 ± 0.03 ^dD^
	28	7.43 ± 0.06 ^eA^	7.10 ± 0.04 ^eB^	6.56 ± 0.08 ^eC^	6.40 ± 0.06 ^eD^	6.23 ± 0.02 ^eE^
*L* value	0	22.18 ± 0.02 ^aA^	22.27 ± 0.04 ^aA^	22.32 ± 0.02 ^aA^	22.41 ± 0.04 ^aA^	22.08 ± 0.04 ^aA^
	7	16.23 ± 0.02 ^bA^	22.04 ± 0.04 ^bE^	22.17 ± 0.06 ^bD^	22.20 ± 0.03 ^bC^	20.75 ± 0.09 ^bB^
	14	15.67 ± 0.03 ^cA^	21.64 ± 0.10 ^cD^	21.76 ± 0.02 ^cD^	21.82 ± 0.03 ^cC^	19.52 ± 0.10 ^cB^
	21	13.41 ± 0.04 ^dA^	21.08 ± 0.04 ^dC^	21.44 ± 0.02 ^dD^	21.64 ± 0.16 ^dE^	18.31 ± 0.12 ^dB^
	28	12.71 ± 0.02 ^eA^	20.71 ± 0.04 ^eD^	21.06 ± 0.03 ^eC^	21.25 ± 0.07 ^eE^	17.34 ± 0.06 ^eB^
*a* value	0	3.19 ± 0.05 ^aA^	3.21 ± 0.03 ^aA^	3.26 ± 0.01 ^aA^	3.26 ± 0.04 ^aA^	3.29 ± 0.02 ^aA^
	7	4.36 ± 0.04 ^bA^	4.08 ± 0.04 ^bB^	3.77 ± 0.08 ^bC^	3.67 ± 0.04 ^bC^	3.43 ± 0.05 ^bD^
	14	5.43 ± 0.10 ^cA^	4.38 ± 0.05 ^cB^	4.29 ± 0.04 ^cC^	4.11 ± 0.04 ^cD^	3.74 ± 0.11 ^cE^
	21	6.08 ± 0.06 ^dA^	5.07 ± 0.05 ^dB^	4.72 ± 0.04 ^dC^	4.37 ± 0.06 ^dD^	3.96 ± 0.09 ^dD^
	28	6.58 ± 0.04 ^eA^	5.55 ± 0.10 ^eB^	5.39 ± 0.09 ^eC^	5.17 ± 0.09 ^eD^	4.25 ± 0.09 ^eE^
*b* value	0	11.18 ± 0.05 ^aA^	11.06 ± 0.03 ^aA^	11.03 ± 0.02 ^aA^	11.06 ± 0.04 ^aA^	11.03 ± 0.02 ^aA^
	7	11.04 ± 0.03 ^bA^	10.44 ± 0.04 ^bD^	10.57 ± 0.06 ^bC^	10.71 ± 0.05 ^bB^	10.78 ± 0.04 ^bB^
	14	9.46 ± 0.13 ^cA^	9.74 ± 0.06 ^cB^	10.00 ± 0.03 ^cC^	10.30 ± 0.12 ^cD^	10.42 ± 0.02 ^cD^
	21	7.22 ± 0.08 ^dA^	7.74 ± 0.09 ^dB^	7.93 ± 0.03 ^dC^	8.11 ± 0.05 ^dD^	8.44 ± 0.08 ^dE^
	28	6.37 ± 0.06 ^eA^	7.14 ± 0.05 ^eB^	7.35 ± 0.09 ^eC^	7.64 ± 0.10 ^eD^	7.76 ± 0.08 ^eD^

Values represent the mean ± SD (*n* = 15). Different lowercase letters represent significant differences (*p* < 0.05) within the same treatment group at each sampling time point as determined by a Duncan’s multiple range test (DMRT) and run vertically within each treatment group. Uppercase letters represent significant differences between different treatment groups at a specific timepoint, as determined by a DMRT ran horizontally across treatments at each sampled timepoint in each of the measured parameters.

**Table 2:** Effect of different HVEF (600 kV/m) treatment times (30, 60, 90, and 120 min) on pectin content and the activity of pectin-degrading enzymes (polygalacturonase PG and pectin methylesterase PME) in tomatoes stored for 28 days at 4 °C.

	Storage Time (Days)	Control Group	High-Voltage Electrostatic Field (HVEF) Treatment with 600 kV/m			
			30 min	60 min	90 min	120 min
Pectin content (%)	0	4.68 ± 0.03 ^aA^	4.68 ± 0.03 ^aA^	4.73 ± 0.01 ^aA^	4.70 ± 0.03 ^aA^	4.71 ± 0.02 ^aA^
	7	4.38 ± 0.03 ^bA^	4.55 ± 0.04 ^bB^	4.57 ± 0.02 ^bB^	4.67 ± 0.02 ^aC^	4.66 ± 0.02 ^bC^
	14	3.62 ± 0.05 ^cA^	4.23 ± 0.04 ^cB^	4.37 ± 0.05 ^cC^	4.46 ± 0.04 ^bC^	4.54 ± 0.02 ^cC^
	21	3.27 ± 0.06 ^dA^	4.06 ± 0.05 ^dB^	4.24 ± 0.03 ^dC^	4.36 ± 0.04 ^cD^	4.43 ± 0.02 ^dD^
	28	2.56 ± 0.07 ^eA^	3.77 ± 0.06 ^eB^	3.92 ± 0.03 ^eC^	4.04 ± 0.03 ^dD^	4.15 ± 0.04 ^eE^
Polygalacturonase (PG) activity (U/mg protein)	0	4.39 ± 0.03 ^aA^	4.44 ± 0.03 ^aA^	4.40 ± 0.02 ^aA^	4.38 ± 0.04 ^aA^	4.40 ± 0.02 ^aA^
	7	7.21 ± 0.06 ^bA^	5.94 ± 0.07 ^bB^	5.40 ± 0.02 ^bC^	4.86 ± 0.13 ^bD^	4.34 ± 0.04 ^bE^
	14	9.86 ± 0.04 ^cA^	7.10 ± 0.06 ^cB^	6.64 ± 0.06 ^cC^	4.91 ± 0.07 ^cD^	4.60 ± 0.06 ^cE^
	21	7.37 ± 0.05 ^dA^	6.79 ± 0.07 ^dB^	5.73 ± 0.02 ^dC^	5.21 ± 0.04 ^dD^	5.08 ± 0.04 ^dE^
	28	6.28 ± 0.03 ^eA^	5.89 ± 0.03 ^eB^	4.77 ± 0.06 ^eC^	4.92 ± 0.04 ^eD^	5.04 ± 0.04 ^eE^
Pectin methylesterase (PME) activity (U/mg protein)	0	0.03 ± 0.02 ^aA^	0.02 ± 0.01 ^aA^	0.03 ± 0.01 ^aA^	0.02 ± 0.01 ^aA^	0.03 ± 0.01 ^aA^
	7	0.16 ± 0.02 ^bA^	0.12 ± 0.01 ^bB^	0.09 ± 0.01 ^bC^	0.05 ± 0.01 ^bD^	0.05 ± 0.01 ^bD^
	14	0.26 ± 0.04 ^cA^	0.25 ± 0.02 ^cA^	0.19 ± 0.01 ^cB^	0.15 ± 0.01 ^cB^	0.14 ± 0.02 ^cB^
	21	7.37 ± 0.05 ^dA^	0.30 ± 0.01 ^dB^	0.24 ± 0.02 ^dC^	0.19 ± 0.01 ^dD^	0.17 ± 0.02 ^dD^
	28	6.28 ± 0.03 ^eA^	0.39 ± 0.03 ^eB^	0.31 ± 0.02 ^eC^	0.24 ± 0.02 ^eD^	0.22 ± 0.02 ^eD^

Values represent the mean ± SD (*n* = 15). Different superscript lowercase letters represent significant differences (*p* < 0.05) between timepoints within the same treatment group as determined by a Duncan’s multiple range test (DMRT). Superscript uppercase letters represent significant differences between different treatment groups at a specific timepoint.

**Table 3:** Effect of different HVEF (600 kV/m) treatment times (30, 60, 90, and 120 min) on total phenolic content (TPC), total flavonoid content (TFC), ascorbic acid, and lycopene in tomatoes stored for 28 days at 4 °C.

	Storage Time (Days)	Control Group	High-Voltage Electrostatic Field (HVEF) Treatment with 600 kV/m			
			30 min	60 min	90 min	120 min
Total phenolic content (TPC, mg GAE/g)	0	57.25 ± 0.09 ^aA^	57.26 ± 0.03 ^aA^	57.26 ± 0.05 ^aA^	57.28 ± 0.04 ^aA^	57.22 ± 0.06 ^aA^
	7	58.28 ± 0.04 ^aA^	57.86 ± 0.06 ^bB^	57.69 ± 0.03 ^bBC^	57.60 ± 0.08 ^bC^	57.45 ± 0.04 ^bD^
	14	56.01 ± 0.62 ^cA^	58.18 ± 0.09 ^cB^	58.13 ± 0.06 ^cB^	58.20 ± 0.06 ^cB^	58.20 ± 0.05 ^cB^
	21	53.20 ± 0.08 ^dA^	57.28 ± 0.05 ^aB^	57.48 ± 0.06 ^aC^	57.60 ± 0.07 ^bD^	57.78 ± 0.06 ^dE^
	28	49.28 ± 0.05 ^eA^	55.18 ± 0.06 ^dB^	56.28 ± 0.04 ^dC^	56.56 ± 0.12 ^dD^	56.69 ± 0.06 ^eD^
Total flavonoids content (TFC, mg QE/g)	0	6.70 ± 0.06 ^aA^	6.75 ± 0.03 ^aA^	6.72 ± 0.03 ^aA^	6.71 ± 0.04 ^aA^	6.68 ± 0.03 ^aA^
	7	5.45 ± 0.02 ^bA^	6.24 ± 0.05 ^bB^	6.32 ± 0.03 ^bC^	6.44 ± 0.03 ^bD^	6.59 ± 0.03 ^bE^
	14	4.62 ± 0.03 ^cA^	5.54 ± 0.10 ^cB^	5.83 ± 0.06 ^cC^	5.88 ± 0.06 ^cC^	5.91 ± 0.03 ^cC^
	21	4.29 ± 0.02 ^dA^	5.14 ± 0.02 ^dB^	5.27 ± 0.06 ^dC^	5.40 ± 0.06 ^dD^	5.54 ± 0.06 ^dE^
	28	3.80 ± 0.05 ^eA^	4.58 ± 0.09 ^eB^	4.81 ± 0.05 ^eC^	5.07 ± 0.05 ^eD^	5.18 ± 0.04 ^eE^
Ascorbic acid (mg/100 g)	0	32.18 ± 0.08 ^aA^	32.21 ± 0.04 ^aA^	32.36 ± 0.04 ^aA^	32.29 ± 0.15 ^aA^	32.34 ± 0.08 ^aA^
	7	27.37 ± 0.05 ^bA^	29.07 ± 0.06 ^bB^	29.63 ± 0.04 ^bC^	30.11 ± 0.09 ^aD^	30.75 ± 0.12 ^bE^
	14	23.12 ± 0.07 ^cA^	27.43 ± 0.09 ^cB^	28.15 ± 0.07 ^cC^	28.75 ± 0.07 ^bD^	29.10 ± 0.07 ^cE^
	21	18.57 ± 0.16 ^dA^	20.12 ± 0.03 ^dB^	22.54 ± 0.11 ^dC^	24.63 ± 0.10 ^cD^	26.46 ± 0.06 ^dE^
	28	12.30 ± 0.23 ^eA^	15.41 ± 0.07 ^eB^	16.65 ± 0.07 ^eC^	17.80 ± 0.16 ^dD^	18.31 ± 0.13 ^eE^
Lycopene (mg/100 g)	0	17.38 ± 0.06 ^aA^	17.40 ± 0.04 ^aA^	17.45 ± 0.02 ^aA^	17.42 ± 0.02 ^aA^	17.46 ± 0.02 ^aA^
	7	16.25 ± 0.03 ^bA^	16.80 ± 0.07 ^bB^	16.85 ± 0.06 ^bB^	17.14 ± 0.03 ^bC^	17.09 ± 0.07 ^bC^
	14	15.34 ± 0.11 ^cA^	15.72 ± 0.04 ^cB^	16.06 ± 0.03 ^cC^	16.07 ± 0.05 ^cC^	16.51 ± 0.08 ^cD^
	21	13.14 ± 0.10 ^dA^	13.56 ± 0.06 ^dB^	14.67 ± 0.05 ^dC^	14.72 ± 0.04 ^dC^	15.18 ± 0.06 ^dD^
	28	12.24 ± 0.14 ^eA^	12.62 ± 0.07 ^eB^	13.13 ± 0.10 ^eC^	13.63 ± 0.08 ^eD^	14.20 ± 0.05 ^eE^

Values represent the mean ± SD (*n* = 15). Different superscript lowercase letters represent significant differences (*p* < 0.05) between timepoints within the same treatment group as determined by a Duncan’s multiple range test (DMRT). Superscript uppercase letters represent significant differences between different treatment groups at a specific timepoint.

## Data Availability

The original contributions presented in the study are included in the article, further inquiries can be directed to the corresponding author.
